# Adipsin, MIP-1b, and IL-8 as CSF Biomarker Panels for ALS Diagnosis

**DOI:** 10.1155/2018/3023826

**Published:** 2018-10-10

**Authors:** Maria Teresa Gonzalez-Garza, Hector Ramon Martinez, Delia E. Cruz-Vega, Martin Hernandez-Torre, Jorge E. Moreno-Cuevas

**Affiliations:** ^1^Tecnologico de Monterrey, Ave. Morones Prieto 3000, Escuela de Medicina y Ciencias de la Salud, Monterrey, NL, 64710, Mexico; ^2^Tecnologico de Monterrey, Batallon de San Patricio 112, Instituto de Neurologıa y Neurocirugıa, Centro Medico Zambrano Hellion, San Pedro Garza García, NL, 66278, Mexico

## Abstract

Amyotrophic lateral sclerosis (ALS) is an aggressive neurodegenerative disorder that selectively attacks motor neurons in the brain and spinal cord. Despite important advances in the knowledge of the etiology and progression of the disease, there are still no solid grounds in which a clinician could make an early objective and reliable diagnosis from which patients could benefit. Diagnosis is difficult and basically made by clinical rating scales (ALSRs and El Escorial). The possible finding of biomarkers to aid in the early diagnosis and rate of disease progression could serve for future innovative therapeutic approaches. Recently, it has been suggested that ALS has an important immune component that could represent either the cause or the consequence of the disease. In this report, we analyzed 19 different cytokines and growth factors in the cerebrospinal fluid of 77 ALS patients and 13 controls by decision tree and PanelomiX program. Results showed an increase of Adipsin, MIP-1b, and IL-6, associated with a decrease of IL-8 thresholds, related with ALS patients. This biomarker panel analysis could represent an important aid for diagnosis of ALS alongside the clinical and neurophysiological criteria.

## 1. Introduction

Amyotrophic lateral sclerosis (ALS) is a neurodegenerative disease characterized by progressive and selective death of upper and lower motor neurons, in the cerebral cortex and spinal cord. To date, there is no effective treatment for this disease or known etiology. Since the identification of SOD1 as a causative gene of ALS, over the past two decades, at least 30 genes have been identified to be associated with ALS. Unlike familial ALS, the causes of sporadic ALS, which accounts for the majority of ALS cases (90–95%), remain unclear [1].

Recently, it has been suggested that ALS could be an autoimmune disease. The increase in activated microglia/macrophages, reactive astrocytes, and dendritic cells found in the postmortem brain and spinal cord of ALS patients supports the concept that an immune-mediated inflammatory process may contribute to ALS pathogenesis that includes proinflammatory cytokine increase in serum and cerebrospinal fluid (CSF) [2–5]. It has also been described that serum from ALS patients induces motor neuron death *in vitro* and *in vivo* in healthy mice yielding deterioration of motor neurons in the spinal cord and alters ion channel expression of the Na(v)1.6 and K(v)1.6 channels in newborn rat spinal motor neurons [6–9]. These recent observations suggest that a toxic event and primary or consequent immune response may eventually induce an apoptotic death of motor neurons. At the present time, these reports do not clarify whether inflammatory processes precede disease onset or result from it [10]. However, they suggest that an inflammatory activity may be present early in ALS and, according to Majoor-Krakauer et al. [11], it could trigger a catastrophic cascade of events leading toward selective motor neuron death in genetically susceptible subjects.

Multiplex cytokine analysis on the CSF of 41 ALS patients showed an increment of IL-10, IL-6, GM-CSF, IL-2, and IL-15 versus the concentrations of these cytokines in the CFS of subjects with other neurological diseases. Also, the expression of IL-8 was higher in those patients with lower levels of physical function [10]. The increase of proinflammatory cytokines has been correlated with increases in activation of microglia/macrophages, reactive astrocytes, and dendritic cells [2] that supports an inflammatory process occurring either at the initiation or at the progression of the disease.

Recently, a cytokine pathway analysis in the CSF of ALS patients report a negative correlation between IL-4 and IL-6 and shorter disease evolution towards death (<12 moths) and a positive correlation on patients with longer more settle disease progression (>12 months) [6, 12]. Adipsin, monocyte chemoattractant protein-1 (MCP-1), and macrophage inflammatory protein-1*β* (MIP-1*β*) increased concentrations have also been reported in the CSF of ALS patients. Although these cytokine CSF levels were higher in patients when compared to controls, no correlation with ALS clinical severity was observed [12, 13]. Nevertheless, disease duration was correlated positively with levels of MCP-1 [4]. The complement system in ALS suggests that activation may precede end-plate denervation in human ALS [14, 15].

ALS diagnosis is a challenging process due to its heterogenic clinical phenotype that overlaps with other neurodegenerative diseases. The diagnosis is based on the El Escorial and Airlie House clinical and neurophysiological criteria [16]. At the present time, there are no reliable biomarker panels that could aid the clinician to establish an early diagnosis, as well as to define prognosis [17]. C-reactive protein, selected interleukins, growth factors, neurofilaments, microRNA, and others, either in serum or in CSF, have been proposed as possible prognosis biomarkers [18–21]. Nevertheless, there is no consensus on which biomarkers are reliable as diagnostic factors in ALS. In this report, we describe a biomarker panel of CSF cytokine concentrations, obtained after applying a tree analysis and a PanelomiX program [22].

## 2. Material and Methods

### 2.1. Patient Samples

Seventy-seven patients between 26 and 77 years of age were recruited (mean age 48.5 ± 11.7) and evaluated for eligibility at the Neurology Service of the Hospital San Jose Tec de Monterrey, Mexico, from June 2005 to December 2010. As for the control group, 13 patients (mean age 39.15 ± 11.32 years) (61% female and 39% male), who underwent a complete neurological evaluation that included a spinal tap for disabling headaches were eventually diagnosed as having a tensional headache. All had normal CSF and head magnetic resonance imaging (MRI). The Ethics and Research Committees of Hospital San Jose and Medicine School from the Tecnológico de Monterrey approved the protocol, and all the participating patients and controls signed an informed consent. CSF was obtained by lumbar puncture, and aliquot of 2 ml from each patient was stored at −80°C.

### 2.2. Cytokine Analysis

Cytokine determination was performed by multiplex analysis of undiluted CSF supernatants using the Bio-Plex Human 17-plex panel of cytokines and growth factors (Bio-Rad; Hercules, CA) (Table 1). To avoid intra- and intertest determination variability, all CSF samples were analyzed at the same time. ALS patients were evaluated by means of the ALS functional rating scale revised (ALSFRS-R) at the time of the lumbar puncture.

### 2.3. Statistical Analysis

CSF concentrations of 19 cytokines of 77 ALS patients and 13 controls were analyzed by a nonparametric Mann-Whitney's *U* two-tailed test to identify differences in central tendencies between groups followed by a Kolmogorov-Smirnov test. Afterward, a linear discriminant analysis and a decision tree were fitted to the complete cases and different measures of classification error were performed. All analysis and graphs were developed using the R programming language (http://www.r-project.org/). For the biomarker panel analysis threshold, PanelomiX, a threshold-based algorithm, was applied (http://www.panelomix.net) [22].

## 3. Results

**Figure 1:**
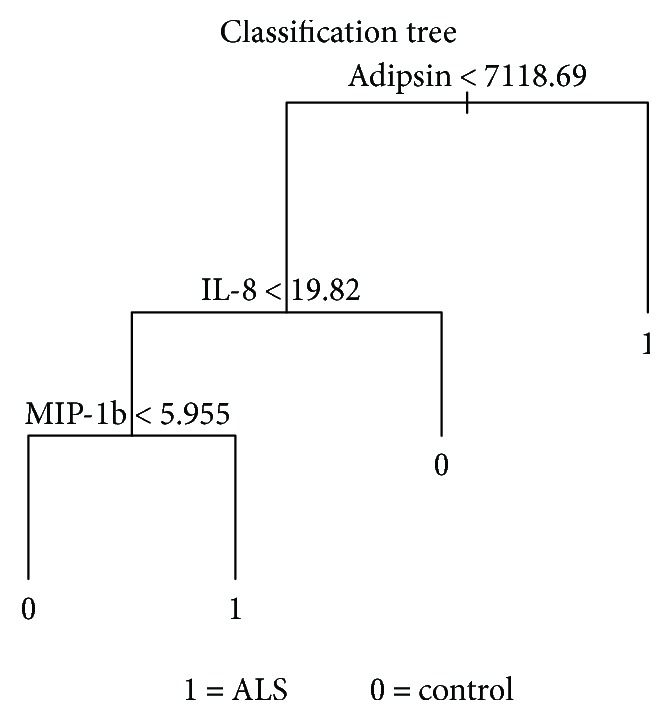
Decision trees displaying the partitioning of the original space into subregions pertaining to one particular group.

Applied classification tree analysis shows adipsin as the first filter. Levels of this protein greater than 7118 ng must likely define the sample as one coming from an ALS patient. On this sheet, 61 patients from 77 (79%) were detected. None of the controls presented such high levels. The second filter was IL-8, which was defined as this cytokine presenting levels under 19.82 ng. On the third filter, MIP-1b was established to be higher than 5.95 ng to confirm it as probably belonging to an ALS patient (Figure 1).

**Figure 2:**
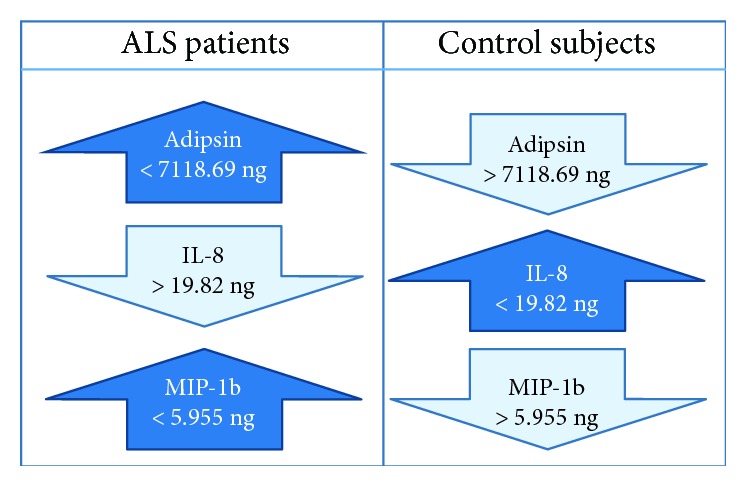
Comparing concentration of adipsin, IL-8, and MIP-1b after classification by decision tree analysis applied to CSF cytokine on the ALS group and the control group.

The applied decision tree to cytokine concentration on the CSF of the control group shows positive levels of adipsin; it must be lower than 7118.49 pg/ml with levels of IL-8 pg/ml higher than 19.82 pg/ml and concentrations of MIP-1b under 5.95 pg/ml.  Figure 2.

This technique yielded an accuracy in the prediction of 98.7%, with a sensitivity of 100% and specificity of 91.6% when using all of the data; the resulting differences are shown in Figure 2. However, when performing fourfold cross-validation, the prediction error rate was greater, resulting in an accuracy of 75.32%, the sensibility of 81.53%, and specificity of 41.6%. When employing leave-one-out cross-validation, the accuracy and sensitivity were improved to 89.6% and 95.38%, respectively, although the specificity remained low at 58.33%.

Results generated with PanelomiX algorithm analysis show very similar results: the same proteins and their thresholds were positive as markers for ALS (Table 2). In addition to the three previous markers, adipsin, MIP-1b, and IL-8, IL-6 was also detected as a positive marker. ALS outcomes were positive when two of the cytokines coincide with the threshold in Table 2.

**Figure 3:**
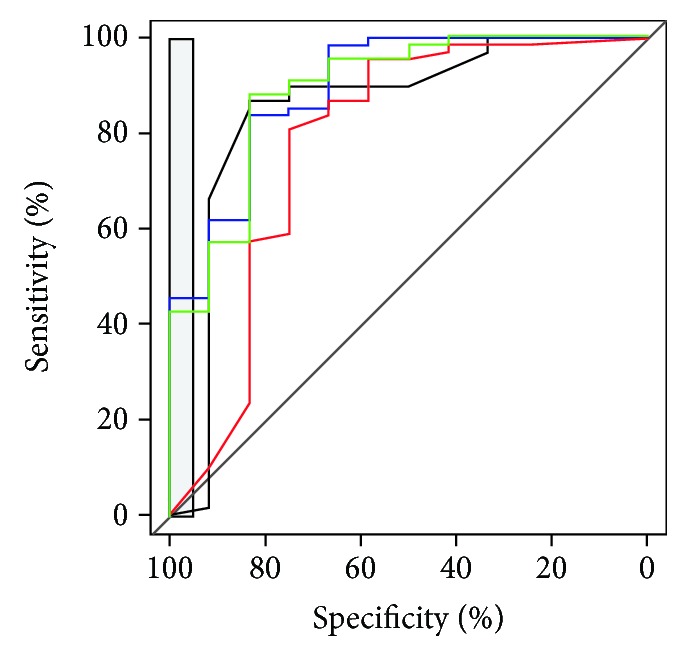
ROC curves showing the comparison with other standard combination methods. Black: PanelomiX; blue: logistic regression; green: SVM; red: recursive partitioning (decision trees).

ROC curves obtained with four standard methods: PanelomiX algorithm, logistic regression, support vector machine (SVM), and decision tree, are shown in Figure 3. In there, we observed that the best results were obtained from PanelomiX and decision tree (Table 3).

## 4. Discussion

The search for possible markers for the diagnosis of ALS included cytokines, growth factors, specific neuronal proteins, and specific mutations [18–21]. However, to date, there is no reliable marker. In this work, we propose the combination of more than one marker that allows us to diagnose ALS with an acceptable sensitivity and specificity. The analysis of the concentrations of a panel of cytokines and their correlation between them allowed to determine that using the decision tree in patients with ALS had low values of IL-8 and high values of MIP-1b and adipsin. PanelomiX algorithm also show a threshold for adipsin, MIP-b1, IL-8, and IL-6 as markers for ALS. This program has shown to be useful to create panels of biomarkers by applying the interactive combination of biomarker and threshold (ICBT) method. The proposed combination model has been demonstrated to be advantageous for predicting the outcome in patients with aneurysmal subarachnoid haemorrhage [22] and prognosis in severe traumatic brain injury [23]. Also, it has been applied to discriminate between patients with lung cancer versus smokers [24, 25]. In a previous work, we reported the high values of MIP-1b and adipsin in patients with ALS [12, 13]. Added into this analysis and as a corollary, low values of IL-6 and IL-8 and high values of adipsin and MIP-1b could be taken into account as strong ALS markers. Between those cytokines, IL-8 represents an important factor to follow. Low values of IL-8 were also reported in multiple sclerosis patients [26]; nevertheless, other reports inform high levels of IL-8 in noninflammatory neurological diseases [27–31]. Because IL-8 induces angiogenesis and proliferation, it is possible that by decreasing its expression, it would reflect a poor recovery tissue capacity and faster disease progression [32, 33]. Also, high levels of IL-8 have been related with lower ALSFRS-R scores and as indicator of disease progression [10].

## 5. Conclusion

The analysis of the levels of IL-6, IL-8, MIP-b1, and adipsin could be part of a biomarker panel for the diagnosis of ALS alongside the clinical and neurophysiological criteria. These observations could also be important to a better understanding about the clinical outcome as well as the participation of inflammatory processes in the disease onset.

## Figures and Tables

**Table 1:** Cytokines and growth factor determined by multiplex system.

Interleukin 1 (IL-1)	Interleukin 7 (IL-7)	Interleukin 17 (IL-17)	Macrophage inflammatory protein (MIP-1b)
Interleukin 2 (IL-2)	Interleukin 8 (IL-8)	Granulocyte colony-stimulating factor (G-CSF)	Tumor necrosis factor (TNF*α*)
Interleukin 4 (IL-4)	Interleukin 10 (IL-10)	Macrophage colony-stimulating factor (M-CSF)	Adipsin
Interleukin 5 (IL-5)	Interleukin 12 (IL-12)	Interferon gamma (IFN*γ*)	Monocyte chemoattractant protein-1 (MCP-1)
Interleukin 6 (IL-6)	Interleukin 13 (IL-13)	Monocyte chemotactic and activating factor (MCAF)	

**Table 2:** Positive markers for ALS disease obtained with PanelomiX analysis.

Adipsin	MIP-1b	IL-6	IL-8
**>**	**>**	**>**	**<**
7118.69	5.89	4.59	22.445

**Table 3:** ROC analysis of panel and classical methods (cross-validation).

	% pAUC (95% CL)	% SP (95% CL)	% SE (95% CL)
PanelomiX	0.0 (0.0–4.6)	100.0 (100.0–100.0)	0.0 (0.0–0.0)
Logistic regression	2.3 (1.8–4.5)	100.0 (100.0–100.0)	45.6 (30.9–54.4)
Decision trees	0.2 (0.0.3.8)	100.0 (100.0–100.0)	0.0 (0.0-0.0)
Support vector machines	2.1 (1.6–4.6)	100.0 (100.0–100.0)	42.6 (30.9–54.4)

## Data Availability

The cytokine concentration data used to support the findings of this study are included within the supplementary information file (available here).

## Supplementary Materials

Supplementary Materials Cytokine concentration determined on undiluted CSF supernatants by multiplex analysis of using the Bio-Plex Human 17-plex panel of cytokines and growth factors.
