# Supplementary material for: Adipsin, MIP-1b, and IL-8 as CSF Biomarker Panels for ALS Diagnosis
Source: Dis Markers. 2018 Oct 10;2018:3023826. doi: 10.1155/2018/3023826 (PMC6199888; doi:10.1155/2018/3023826)
Supplement: Supplementary Materials — Cytokine concentration determined on undiluted CSF supernatants by multiplex analysis of using the Bio-Plex Human 17-plex panel of cytokines and growth factors. [file 3023826.f1.pdf]

Grupo	IL-1b (39)	u IL-2 (38)	u IL-4 (52)	u IL-5 (33)	u IL-6 (19)	u IL-7 (74)	u IL-8 (54)	IL-10 (56)	?(p70) (75)	IL-13 (51)	IL-17 (76)	-CSF (57)	I-CSF (34)	IFN-g (21)	ICAF) (53)	IIP-1b (18)	TNF-a (36)	Adipsin	diponectin
1	0.19	0.99	0.31	0.32	1.78	0.0001	11.18	1.54	0.0001	1.12	4.99	17.04	27.09	0.0001	107.05	3.66	0.25	7264.66	2368.86
1	0.34	1.55	0.55	0.28	0.99	0.0001	17.99	1.33	0.0001	0.36	0.0001	10.52	28.56	0.0001	202.08	13.65	1.77	8374.91	3624.71
1	0.13	0.0001	0.24	0.2	0.21	0.0001	1.97	1.89	0.0001	0.45	0.0001	8.11	26.83	0.0001	56.13	10.68	0.0001	1775.03	12723.9
1	0.29	0.0001	0.16	0.45	21.73	0.77	36.21	2.02	0.0001	11.57	0.0001	13.11	26.83	3.36	468.09	19.35	1.77	19967.49	7717.68
1	0.33	0.0001	0.14	0.49	5.53	0.58	30.05	1.82	0.0001	0.28	0.0001	7.13	22.87	0.0001	271.98	15.44	1.77		
1	0.17	0.0001	0.38	0.41	2.05	0.0001	23.55	1.99	0.0001	10.88	0.0001	7.62	23.3	0.0001	143.2	13.68	0.88		
1	0.29	1.45	0.21	0.28	2.51	1.03	10.54	1.42	0.0001	0.53	0.0001	23.36	25.62	0.0001	219.37	10.12	1.77		
1	0.25	0.0001	0.26	0.12	1.52	0.9	12.47	2.55	0.0001	13.51	6.16	6.14	25.28	1.71	206.45	11.1	2.46	17245.56	8577.14
1	0.16	0.37	0.0001	0.3	1.25	0.96	10.99	1.45	0.0001	0.0001	0.0001	6.14	26.4	0.0001	133.54	7.13		38797.99	15838.38
1	0.23	0.0001	0.26	0.18	1.25	0.0001	7.98	0.03	0.0001	0.7	0.0001	4.62	21.32	0.0001	80.35	6.04	2	23052.16	5270.97
1	0.16	0.0001	0.16	0.37	0.0001	0.0001	4.15	0.8	0.0001	0.79	0.78	9.56	21.49	0.0001	73.83	4.67	0.66	1466.62	4264.76
1	0.42	2.78	0.16	0.24	0.66	0.0001	14.01	1.2	0.0001	0.01	0.0001	12.41	24.76	0.0001	96.58	5.3	0.0001	26556.57	14734.59
1	0.18	0.05	0.09	0.28	2.05	0.0001	12.47	1.03	0.0001	2.18	0.0001	8.11	26.66	0.0001	142.01	14.71	4.61	15232.12	2781.68
1	0.19	0.0001	0.41	0.62	4.05	0.38	16.7	1.54	0.0001	0.19	0.0001	2.53	23.81	1.71	297.33	18.84	1.32	5536.57	2152.43
1	0.29	0.0001	0.45	0.32	4.86	0.51	13.94	1.48	0.0001	0.06	0.0001	9.8	26.14	0.0001	369.37	7.76	1.32	18010.53	2368.86
1	0.17	0.0001	0.43	0.18	0.92	0.0001	29.92	1.15	0.0001	0.0001	0.0001	5.39	28.56	0.0001	140.97	10.19	2.23	23280.41	6527.92
1	0.18	0.0001	0.36	0.12	2.18	0.0001	10.99	1.63	0.0001	8.61	0.0001	9.32	22.52	7.75	80.62	8.52	0.88	17158.32	2368.86
1	0.22	0.0001	0.43	0.51	3.85	0.31	29.4	1.48	0.0001	0.0001	0.0001	3.59	18.5	4.89	499.66	9.13	1.32	10397.67	2368.86
1	0.18	0.08	0.21	0.49	2.25	0.11	17.86	1.57	0.0001	0.0001	0.0001	7.13	28.04	0.0001	225.37	5.81	3.4		
1	0.48	0.25	0.31	0.49	4.86	0.0001	22.97	1.82	0.0001	2.5	0.0001	11.47	13.13	0.0001	438.07	15.28	1.77	10992.85	2781.68
1	0.04	2.02	0.41	0.2		0.77	5.3	1.95	0.0001	0.7	0.0001	2.53	35.16	0.0001	81.01	4.95	0.0001	3504.32	2578.25
1	0.25	0.0001	0.16	0.32	4.05	0.0001	22.25	1.89	0.0001	0.19	0.0001	10.52	24.24	0.0001	367.65	12.13	2.93	22840.11	3732.6
1	0.34	0.0001	0.24	0.53	4.05	0.31	20.19	0.8	0.0001	4.01	8.07	7.13	19.1	0.0001	262.07	18	1.32	13245.73	6830.78
1	0.45	0.0001	0.24	0.7	3.85	1.09	37.26	1.99	0.5	5.58	0.0001	20.67	20.64	1.71	436.33	12.09	2.23	24760.13	9961.24
1	0.29	0.0001	0.55	0.28	2.18	1.15	23.03	1.51	0.0001	2.9	0.0001	9.08	23.21	12.42	230.54	15.77	4.12	15180.55	2368.86
1	0.95	0.0001	0.21	0.66	3.11	1.92	67.84	1.51	0.0001	54.76	0.0001	10.99	39.88	0.0001	186.78	24.99	2.23		
1	0.42	0.0001	0.31	0.62	2.05	0.77	23.42	1.54	0.78	0.32	3.41	35.59		7.75	150.34	29.62	8.59	24313.67	8006.94
1	0.27	0.0001	0.45	0.28	3.38	0.9	10.48	1.57	1.31	20.71	0.0001	138.78	21.84	0.0001	158.39	2.72	0.88	10682.71	3173.72
1	0.18	0.0001	0.71	0.78	112.57	0.31	85.03	2.48	0.0001	0.62	0.0001	15.2	10.76	10.46	717.69	17.36	2.46	12126.84	3173.72
1	0.53	0.0001	0.45	0.41	3.98	1.09	47	1.51	0.0001	13.06	0.0001	10.04	6.89	0.0001	368.92	14.05	2.93		
1	0.45	0.0001	0.06	0.53	1.52	4.29	13.56	2.68	0.0001	19.35	0.0001	1.99	12.8	0.0001	216.84	11.72	7.08	27941.38	12080.58
1	0.3	0.99	0.6	0.57	6.21	0.9	29.14	1.95	0.08	1.37	0.0001	28.22	24.59	1.71	270.85	8.16	1.1	13419.88	7129.84
1	0.23	0.0001	0.26	0.32	8.94	0.25	22.77	2.08	0.0001	1.49	0.0001	13.81	21.66	1.71	182.42	8.38	0.45	15477.77	4607.76
1	0.37	0.0001	0.41	0.37	10.31	0.25	22.25	1.33	0.0001	0.19	0.0001	10.04	26.92	0.0001	331.76	7.9	3.4	21118.93	13106.21
1	0.5	0.0001	0.31	0.28	1.98	0.05	6.07	0.8	0.0001	1.69	0.0001	16.12	17.64	6.34	250.11	8.77	3.64	20367.31	4607.76
1	0.23	0.0001	0.11	0.57	2.18	0.38	13.62	1.48	0.0001	0.15	1.32	7.13	21.32	0.0001		11.48	0.66	8906.73	3549.65
1	0.27	0.0001	0.21	0.41	1.38	0.38	12.34	1.23	0.0001	0.45	0.0001	10.52	20.89	0.0001	180.45	7.72	1.32	8280.01	4607.76
1	0.22	0.0001	0.21	0.24	5.13	0.7	13.3	1.09	0.08	1.04	0.0001	20.22	16.96	0.0001	268.81	13.04	1.32	22164.93	5107.72
1	0.29	0.0001	0.67	0.85	2.51	1.15	59.28	2.08	0.64		0.0001	23.36	3.21	0.0001	255.67	9.34	3.88	35252.23	13611.95
1	0.23	0.0001	0.41	0.22	0.0001	0.25	17.35	2.89	0.0001	1.2	0.0001	16.12	17.39	0.0001	149.41	1.91	1.54	24010.6	34379.47
1	0.39	0.0001	0.69	0.2	2.31	0.38	34.7	1.76	0.0001	95.53	0.0001	19.09	20.12	0.0001	251.84	17.49	0.45	30675.97	14113.4
1	0.34	0.0001	0.11	0.41	2.85	0.77	18.12	2.05	0.0001	0.06	0.0001	12.17	33.59	7.75	166.08	12.6	1.32	8280.01	3549.65
1	0.39	0.0001	0.24	0.57	0.14	0.31	21.22	1.18	0.0001	61.74	0.0001	34.09	9.92	10.46	314.72	11.44	5.83	20785.01	7862.67
1	0.18	0.0001	0.74	0.24	1.06	2.42	21.54	1.45	0.0001	0.7	0.0001	29.97	29.77	0.0001	176.62	9.13	2.23	14084.27	7129.84
1	0.45	0.01	0.55	0.45	0.73	0.25	23.29	1.89	0.0001	0.87	0.0001	17.27	20.46	4.89	215.08	10.82	2.23	10113.76	3549.65
1	0.31	0.0001	0.33	0.62	0.47	0.9	24.07	1.6	0.5	18.6	0.0001	15.66	32.55	69.71	313.43	7.5	3.64	20611.72	2781.68
1	0.21	0.0001	0.55	0.37	1.91	1.41	18.77	3.17	0.0001	3.82	0.0001	27.34	23.21	10.46	294.74	13.68	5.59	25481.75	8006.94
1	0.31	0.0001	0.33		2.31	1.03	18.64	1.06	0.0001	2.26	0.0001	15.66	23.21	1.71	227.71	10.16	2.69	16844.23	5270.97
1	0.23	2.33	0.16	0.37	3.85	0.0001	22.97	1.63	0.0001	40.07	0.0001	18.18	18.5	0.0001	230.66	18.26	2	40910.28	6374.97
1	0.53	0.43	0.24	0.74	4.93	1.41	28.68	1.57	0.0001	68.67	0.0001	11.94	15	4.89	312.22	14.65	8.85	21897.54	3912.6
1	0.43	0.61	0.45	0.37	1.65	1.79	12.79	1.95	0.0001	8.34	0.0001	39.02	22.09	0.0001	241.43	8.63	4.61	15103.31	7129.84
1	0.31	1.72	0.38	0.41	4.99	0.9	28.55	1.45	0.08	4.96	0.0001	16.58	21.58	2.56	143.05	22.33	3.4	20152.7	14610.83
1	0.31	0.0001	0.6	0.87	3.11	1.03	20.9	2.05	0.23	1.77	19.95	15.66	19.1	0.0001	231.84	10.3	5.59	23242.29	5107.72
1	0.37	0.0001	0.92	0.64	1.38	1.54	13.49	1.7	0.0001	3.62	0.0001	64.45	20.81	6.34	172.86	19.66	2.69	24329.26	1927.54
1	0.37	0.0001	0.5	0.45	4.25	0.11	23.68	2.02	0.0001	1.08	0.0001	14.74	17.56	10.46	282.12	6.64	1.32	18690.87	4607.76
1	1.36	1.16	0.42	0.38	5.55	5.57	42.44	5.76	2.05	72.94	28.76	59.99	85.77	33.54	372.3	20.01	17	12650.94	21834.67
1	0.15	0.0001	0.35	0.4	8.57	11.58	88.21	7.8	0.56	106.08	3.42	177.33	97.47	12.62	417.09	27.73	8.49	11608.56	18192.46
1	0.49	0.21	0.15	0.26	0.89	0.08	1.93	6.68	0.92	8.87	16.52	11.06	89.64	7.03	142.15	6.65	4.05	894.76	1181.1
1	0.69	0.0001	0.15	0.21	8.8	1.25	4.6	4.28	0.56	1.66	16.14	23.15	48.3	0.0001	110.38	5.62	2.45	4195.84	3229.01
1	0.12	0.0001	0.15	0.21	0.89	5.67	3.88	4.32	0.33	27.86	1.61	1.96	65.12	3.85	201.61	6.38	2.45	1764.47	2170.47
1	0.07	0.0001	0.03	0.21	9.49	4.49	12.51	5.38	0.1	2.03	1.43	19.15	64.57	0.0001	119.05	7.33	2.09	4479.52	3080.08

1	0.15	0.0001	0.0001	0.5	5.49	0.08	11.95	5.22	0.38	0.03	0.18	19.15	52.75	0.0001	305.48	13.54	2.8	6566.9	5517.93
1	0.34	0.0001	0.59	0.67	9.39	4.55	12.23	4.97	1.54	22.95	11.32	45.47	47.74	31.4	294.6	7.77	4.94	6314.33	2485.59
1	0.21	0.0001	0.38	0.65	3.61	13.93	13.14	5.11	1.29	59.88	3.96	67.17	53.3	17.69	459.57	14.41	4.94	7855.56	6925.1
1	0.42	0.0001	0.29	0.3	8.6	4.36	13.63	3.15	0.8	33.96	12.99	48.52	33.31	15.2	428.12	7.77	6.71	5937	4369.03
1	0.33	0.0001	0.22	0.08	1.62	10.79	9.14	4.97	0.68	8.38	12.71	34.47	53.72	11.29	48.82	13.45	3.52	4626.4	1134.92
1	0.47	0.0001	0.15	0.13	2.34	3.96	4.6	2.49	0.33	17.63	14.47	45.86	34.6	0.0001	233.62	6.17	7.78	4494.52	4425.32
1	0.09	0.0001	0.19	0.42	3.55	1.25	17.85	4.39	0.38	0.16	0.71	41.22	53.58	0.0001	289.82	14.62	2.63	7546.74	8891.45
1	0.07	0.0001	0.03	0.52	1.43	14.8	10.26	4.23	0.33	15.8	1.07	136.11	57.72	0.0001	277.32	7.58	2.63	4505.04	5830.37
1	0.12	0.0001	0.05	0.52	4.09	11.23	15.18	4.74	0.33	18.98	0.89	79.38	43.26	0.0001	337.52	18.62	2.63	7573.91	3835.37
1	0.47	0.0001	0.32	0.5	3.17	3.63	12.3	4.48	1.29	7.06	13.17	47	48.58	17.69	236.16	8.2	4.58	6289.34	3601.89
1	1.27	0.0001	0.83	0.73	3.67	4.32	9.56	1.93	4.13	10.29	26.23	40.83	60.74	64.93	237.67	9.73	16.47	3571.69	1504.44
1	0.12	0.0001	0.22	0.54	3.29	7.41	15.32	5.8	0.68	11.66	1.79	151.71	74.62	3.85	314.7	17.46	3.87	6691.36	4359.65
1	0.5	0.0001	0.11	0.21	1.3	0.82	3.09	2.14	0.56	0.03	15.77	15.92	34.74	2.03	309.1	6.17	2.09	3579.64	1264.23
1	0.47	0.0001	0.07	0.21	2.03	0.6	6.03	1.05	0.33	0.0001	12.99	19.6	27.38	0.0001	192.32	4.96	2.09	2791.76	1024.09
1	0.1	0.0001	0.07	0.26	2.8	39.49	17	4.87	0.8	25.53	1.97	311.11	69.2	3.85	359.61	17.11	2.8	2424.68	1781.78
1	0.1	0.0001	0.03	0.48	2.29	42.21	14.13	4.19	0.38	18.44	0.54	119.84	49.41	0.0001	284.27	11.88	2.45	5305.85	5555.76
0	0.33	0.25	0.55	0.41	4.32	1.03	22.64	1.95	0.0001	4.72	0.0001	100.91	18.92	7.05	313.04	11.17	9.87	6972.73	2490.31
0	4.07	0.0001	0.24	0.32	0.86	0.0001	33.06	1.63	0.0001	4.41	0.0001	23.36	18.24	0.0001	69.79	3.15	2.93	1331.99	592.21
0	4.96	0.0001	0.0001	0.0001	1.22	0.36	35.92	0.34	0.0001	0.0001	0.0001	14.77	13.99	0.0001	63.69	2.61	0.68	6474.28	4182.45
0	4.43	0.0001	0.0001	0.0001	1.57	1.48	39.48	0.01	0.0001	0.42	0.0001	3.16	7.95	8.93	75.78	3.02	0.0001	1578.39	770.01
0	0.3	0.0001	0.73	0.65	1.73	2.45	12.37	5.29	1.79	4.05	9.84	17.78	53.72	45.86	143.82	5.74	6.71	3844.1	3732.59
0	0.54	0.0001	0.31	0.34	1.41	3.79	7.3	2.95	0.92	29.24	12.89	10.04	42.41	17.69	205.24	4.48	5.29	3290.98	4415.94
0	0.24	0.0001	0.19	0.26	1.09	2.04	9.56	4.97	0.56	1.18	9.47	7.93	38.45	7.03	185.61	4.62	3.52	3288.43	2875.51
0	0.15	0.0001	0.15	0.3	1.38	16.13	12.09	5.01	0.44	34.04	0.71	14.01	40.43	3.85	235.9	4.69	2.98	3039.92	3173.14
0	0.09	0.0001	0.03	0.3	1.62	7.8	9.56	4.69	0.21	5.38	0.89	9	53.58	0.0001	218.07	5.55	2.45	3290.98	4331.52
0	0.09	0.0001	0.11	0.3	3.67	7.03	3.74	6.3	0.21	0.99	1.97	11.06	78.53	0.0001	251.52	3.82	3.16	2868.8	1171.87
0	0.12	0.0001	0.11	0.34	3.2	2.69	154.59	9.49	0.56	30.51	1.61	7.93	65.12	3.85	83.48	9.71	2.8	5324.36	6657.69
0	0.1	0.0001	0.03	0.42	3.26	2.28	158.44	11.51	0.8	27.22	2.15	9	62.52	3.85	85.43	10.88	3.16	6001.35	6638.61
0	1.285	0.020925	0.204183	0.30335	2.110833	3.923342	41.5625	4.511667	0.457533	11.84668	3.2942	19.07917	41.155	8.175867	160.9475	5.786667	0.0001		
